# A Novel Multi-Mode Thermal Therapy for Colorectal Cancer Liver Metastasis: A Pilot Study

**DOI:** 10.3390/biomedicines10020280

**Published:** 2022-01-26

**Authors:** Wentao Li, Yue Lou, Guangzhi Wang, Kangwei Zhang, Lichao Xu, Ping Liu, Lisa X. Xu

**Affiliations:** 1 Department of Interventional Radiology, Fudan University Shanghai Cancer Center, Shanghai 200030, China; wentaoli@fudan.edu.cn (W.L.); lichaoxu@shca.org.cn (L.X.); 2 Department of Oncology, Shanghai Medical College, Fudan University, Shanghai 200030, China; 3 Med-X Research Institute, School of Biomedical Engineering, Shanghai Jiao Tong University, Shanghai 200030, China; LY_017082910045@sjtu.edu.cn (Y.L.); guangzhiwang@sjtu.edu.cn (G.W.); p916740699@alumni.sjtu.edu.cn (K.Z.)

**Keywords:** multi-mode thermal therapy, colorectal cancer liver metastasis, thermal immune response

## Abstract

A novel multi-mode thermal therapy was developed for local tumor ablation and the systemic stimulation of anti-tumor immunity, consisting of a rapid liquid nitrogen freezing, and followed by the radiofrequency heating of target tumor tissue. This pilot study aimed to compare the therapeutic effects of the new therapy with conventional radiofrequency ablation (RFA) on patients with colorectal cancer liver metastasis (CRCLM). From August 2016 to September 2019, thirty-one patients with CRCLM received either multi-mode thermal therapy (*n* = 17) or RFA (*n* = 14). Triphasic contrast-enhanced magnetic resonance imaging (MRI), routine blood tests, and peripheral blood immune responses were evaluated before the treatment and in 1, 3, 6, and 12 months after. Local tumor response and progression-free survival (PFS) were assessed using the Kaplan-Meier method, and pre- and post-treatment immune cell counts were analyzed using Mann-Whitney U and Wilcoxon tests. A significantly longer PFS was observed in the multi-mode thermal therapy group in comparison to that of the conventional RFA group (median, 11.4 versus 3.4 months, *p* = 0.022). It was found that multi-mode therapy induced the functional maturation of dendritic cells, promoted CD4^+^ T cell-mediated antitumor responses, and decreased regulatory T cells, contributing to better therapeutic efficacy in CRCLM patients.

## 1. Introduction

The control of metastatic tumors is a long-sought goal in cancer therapy. Accumulating evidence has shown that the induction of tumor-specific adaptive immunity is essential for the long-term control of cancer. Local ablation is recommended as a radical therapy for hepatic malignancy by many guidelines [1]. Conventional radiofrequency ablation (RFA, temperatures > 60 °C) and cryosurgery (temperatures of −20 °C to −40 °C) have been used for local tumor therapy in the clinic, while tumor recurrence remains a challenge. Anti-tumor immunological responses were observed occasionally [2,3,4,5]. To enhance the stimulation of systemic antitumor immunity, a novel multi-mode thermal therapy was developed in our previous studies [6,7]. This multi-mode approach involves the precise control of a hybrid thermal process characterized by a rapid liquid nitrogen cooling followed by a rapid radiofrequency heating. Through this procedure, while the tumor center was completely coagulated by high temperatures, a peripheral tumor zone was created, in which tumor cells and vasculature were completely broken under sub-lethal temperatures to allow the maximal release of tumor-associated molecules and antigens in situ.

A better therapeutic effect of the multi-mode therapy was proven in animal studies using the subcutaneous 4T1 murine mammary carcinomas and experimental lung metastases of murine B16F10 melanoma, achieving long-term survival rates of over 70% and 80%, respectively [8,9]. Long-lasting anti-tumor efficacy was found to be dependent on the CD4^+^ T helper type (Th) 1-dominant memory response after the therapy [9]. Elevated heat shock protein 70 release was observed at the tumor site and in the circulation, and dendritic cell (DC) maturation elicited [8]. The inflammatory cytokine, interleukin (IL)-6 was also significantly upregulated immediately after the therapy [10]. These factors could create a favorable environment to systematically elicit anti-tumor immune responses to kill tumor cells.

In this study, we conducted a pilot study to investigate the effect of the multi-mode thermal therapy in patients with colorectal liver metastases (CRCLM). The stimulation of antitumor immunity was evaluated through peripheral blood tests before and after the therapy.

## 2. Materials and Methods

### 2.1. Patient Selection

The clinical study was approved by the Ethics Committee of the Fudan University Shanghai Cancer Center (No. 1604159-3-1605&1604159-3-1606) and complied with the Declaration of Helsinki. Patients who met the eligibility criteria were recruited into the study from August 2016 to September 2019. Written informed consent was provided by all subjects.

Patient Inclusion Criteria: Patients (ages, 18–70 years) with histologically confirmed hepatic colorectal metastasis (CRCLM) who previously underwent a radical resection of the primary lesion and had no local recurrence or extrahepatic metastasis were included. They had to have at least one measurable liver metastasis but no more than five lesions, which should be smaller than 5.0 cm in diameter [11].

Patient Exclusion Criteria: Patients with severe disorders of the heart, lung, liver, or kidney function, irreversible coagulation disorder, or other uncontrolled diseases, including hypertension or diabetes, active infection, mental illness, or a social condition that may affect the subject’s compliance, were excluded. Pregnant or lactating women were excluded [11].

A total of thirty-one patients with CRCLM were recruited to participate in this pilot study. The patients were randomly assigned to receive multi-mode thermal therapy or conventional RFA. All patients received a triphasic contrast-enhanced magnetic resonance imaging (MRI) of the abdomen prior to randomization. Electrocardiography (ECG), routine blood tests, and peripheral blood immune status assessment were performed on admission, and other diagnostic procedures (PET-CT, et al.) were performed when indicated.

### 2.2. Clinical Treatment

**Figure 1:**
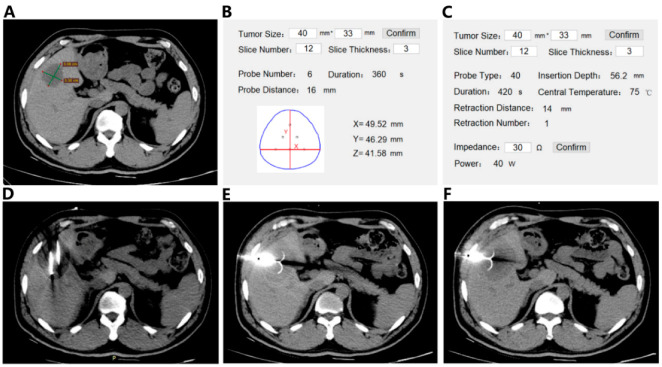
Operational procedure of the multi-mode thermal therapy: a representative case. (**A**) The axial CT imaging showing a 40 mm liver tumor in the patient; (**B**) the plan for freezing; (**C**) the plan for heating; (**D**) freezing process using seven cryo-probes (CryoHitTM, GALIL Medical, Israel). The low signal area near the cryo-probes indicating the formation of ice ball; (**E**) the first step of radiofrequency ablation (RFA), the probe with 4.0 cm active tip (MedSphere, Shanghai, China) inserted to the bottom of the tumor; (**F**) the staged retreat heating process to make the heating range cover the entire tumor.

Multi-mode Thermal Therapy: The new multi-mode thermal therapy procedure consisted of a sequential rapid freezing, natural thawing, and radio-frequency (RF) heating of the target tumor tissue. Cryoprobes (CryoHitTM, GALIL Medical, Yokneam, Israel) and an expandable hook-shaped RF probe (MedSphere, Shanghai, China) were used in this clinical study. For surgical planning, a commercial three-dimensional segmentation software (HG-mediGPS-I, Hokai Medical Instrument Co., Ltd., Zhuhai, China) was used to extract the geometry and relative position of the tumor, liver, ribs, and other organs based on MRI images. The segmented geometric model was then imported into the finite element analysis software (COMSOL Multiphysics 5.2) for computation. In the freezing step, the number of cryo-probes, relative distances, freezing power, and time were calculated based on the bio-heat transfer model to assure rapid freezing and to allow the ice ball to cover the entire tumor for 10 min. Natural thawing was allowed for 5–10 min. In the subsequent RF heating, the probe insertion depth, center temperature, heating power and time, and number of staged retreating and distances were calculated based on our previously developed model accounting for the pre-frozen tissue property changes [6,12]. The total RF heating period ranged from 10–15 min, depending on the tumor size. Figure 1 shows the operational procedure of the multi-mode thermal therapy under CT (64-slice CT scanner, Brilliance CT, Philips, Netherland) guidance.

Conventional RFA: RFA was performed using eight expandable hook-shaped probes (17 gauge, MedSphere, Shanghai, China). All conventional RFAs were performed according to the manufacturer’s protocol.

### 2.3. Follow-Ups

Follow-ups were performed after the multi-mode therapy or conventional RFA. All patients were evaluated by triphasic contrast-enhanced MRI (3T MRI scanner, Skyra, Siemens Healthcare, Germany) before or in 1, 3, 6, and 12 months after the treatments. The MR images in 1 month after treatments were regarded as the baseline assessment. Local tumor response was classified either as a complete response (CR), partial response (PR), stable disease (SD), or progressive disease (PD), according to the modified Response Evaluation Criteria in Solid Tumors. Progression-free survival (PFS) was also assessed. Peripheral blood immune response assessments and routine blood tests were performed before or on day 3, and in 1, 3, 6, and 12 months after treatments. Among the routine blood tests, the lymphocyte, neutrophil, and monocyte counts, and the neutrophil/lymphocyte ratio (NLR) and monocyte/lymphocyte ratio (MLR) were assessed.

### 2.4. Immune Cell Analyses

Reagents and Antibodies: Flow cytometry was performed on a BD FACS Aria-II flow cytometer (BD Biosciences, New Jersey, USA) and analyzed by FACS Diva and FlowJo software. Reagents and antibodies used in the flow cytometric analysis are shown in Table 1 and Table 2.

Isolation of Peripheral Blood Mononuclear Cells: Peripheral blood mononuclear cells were isolated by Ficoll gradient centrifugation (Dakewei Biotec, Shenzhen, China). For the assessment of regulatory T cells (Tregs, CD3^+^CD4^+^CD25^+^Foxp3^+^), the cells were treated with True-Nuclea Transcription Factor Buffer Set (Biolegend, San Diego, CA, USA), according to the manufacturer’s protocol. For the assessment of cytokines (CD4^+^, CD8^+^ T cells, and DCs), the cells were stimulated with a Cell Activation Cocktail (containing brefeldin A, 20 μg/mL) for 4 h, followed by FcγR blockade and surface staining. For the intracellular staining, cells were fixed and permeabilized according to the manufacturer’s instructions. The subsets of CD4^+^ T cells were identified as: Th1 (CD3^+^CD4^+^IFN-γ^+^), Th2 (CD3^+^CD4^+^IL-4^+^), and Th17 (CD3^+^CD4^+^IL-17A^+^). The expression of interferon-γ (IFN-γ), perforin, and granzyme-B were assessed in CD3^+^CD8^+^ T cells. Mature DCs were identified as being CD11c^+^CD86^+^, and the expression of IL-12 and IL-10 were assessed in the CD11c^+^CD86^+^ DCs. The gating strategy was shown in Figure S1.

### 2.5. Statistical Analysis

All data were shown as means ± standard deviations (SDs) for each time point. The two-tailed Mann-Whitney U-test and Wilcoxon test were respectively used to determine differences between groups, or different time points in the same group, using GraphPad Prism 7 (https://www.graphpad.com/scientific-software/prism/, accessed on 21 January 2022). PFS was calculated using the Kaplan-Meier method and compared using the log-rank test with IBM SPSS Statistics for Windows, version 25 (IBM Corp., Armonk, NY, USA). *p* < 0.05 was considered statistically significant.

## 3. Results

### 3.1. Patients and Treatments

Thirty-one patients with CRCLM, 17 in the multi-mode therapy group, and 14 in the conventional RFA group, were included in this pilot study. Patients’ characteristics can be found in Table 3, and there were no significant differences between the two groups. It is seen that most of the enrolled patients suffered from disease progression who had undergone at least two lines of systemic chemotherapy.

In this study, all the treatments were successfully carried out by following the procedures described in Section 2 of Materials and Method. The new multi-mode therapy was found well-tolerated in all patients in the group without major complications.

### 3.2. MRI Features and PFS Assessment after Multi-Mode Therapy

**Figure 2:**
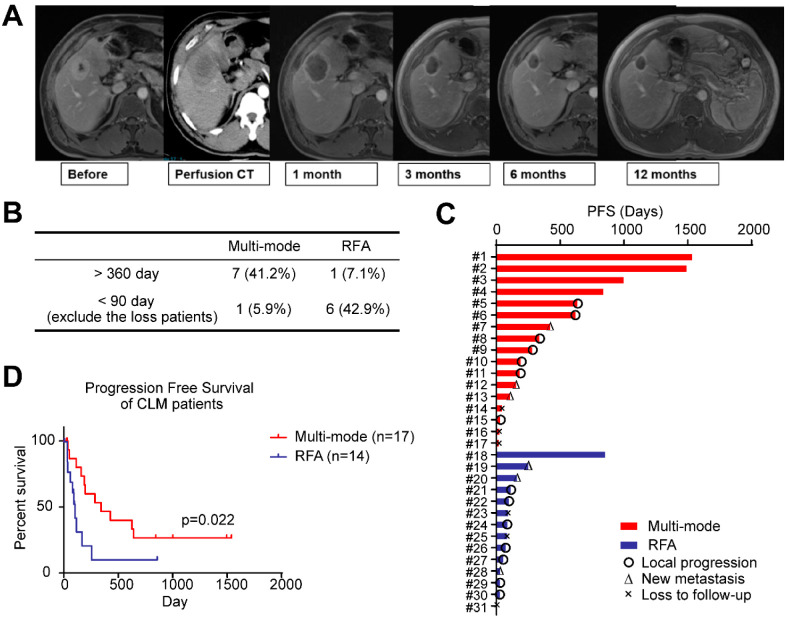
Multi-mode thermal therapy prolongs the progression-free survival (PFS) in patients with malignant hepatic tumors. (**A**) Imaging assessment of a patient treated with multi-mode thermal therapy; (**B**) summary of the long-term and short-term PFS patients in the multi-mode and radiofrequency ablation (RFA) groups; (**C**) summary of the follow-up status and the results of follow-up termination of all patients; (**D**) Kaplan-Meier curves for PFS. The *p*-value indicating the between-group differences.

One month after the multi-mode therapy, CR was achieved in all treated lesions based on MRI. Figure 2A showed the representative MRI images of one patient taken in follow-ups. The sharp boundary of the treated region was clearly visible and the lesion size decreased by time, indicating a precise control of the local tumor ablation.

In result, 7/17 patients in the multi-mode therapy group exhibited no progression for more than one year (1-year PFS rate: 41.2%), with a median PFS time of 11.4 months (range from 1.0 to 51.4 months). On the other hand, only 1/14 patient in the conventional RFA treatment achieved no progression for more than one year (1-year PFS rate: 7.1%), with a median PFS time of 3.4 months (range from 1.0 to 28.6 months) (Figure 2B,C). The percentile of patients with PFS of shorter than 3 months was lower in the multi-mode group than that in the RFA group (5.9% versus 42.9%, Figure 2B,C). Multi-mode therapy resulted in significantly better PFS than the conventional RFA (Figure 2D, *p* = 0.022).

### 3.3. Multi-mode Therapy Altering Preoperative Immunosuppression

To investigate whether the status of preoperative patients could influence the therapeutic efficacy, routine blood parameters before treatments were analyzed. High levels of NLR and MLR were reported to be immunosuppressive markers and to be predictors of CRCLM recurrence following RFA [13,14]. In this study, no 2.4

**Figure 3:**
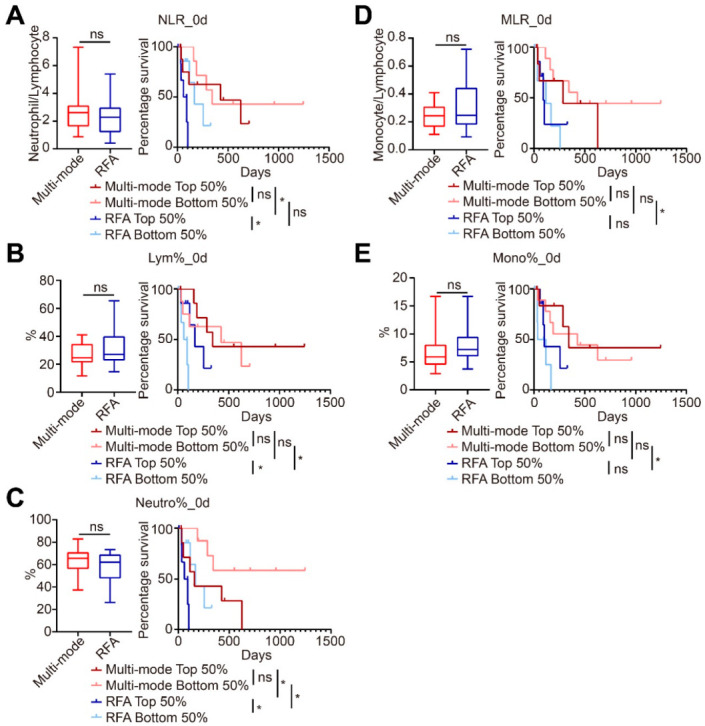
Multi-mode therapy reversed the patients’ preoperative status with improved prognosis. The basic levels and the prognostic significance of the neutrophil-lymphocyte ratio (NLR) (**A**), lymphocytes (**B**), neutrophils (**C**), monocyte-lymphocyte ratio (MLR) (**D**), and monocytes (**E**) in the multi-mode therapy and conventional radiofrequency ablation (RFA) groups. * *p* < 0.05, the *p*-value indicating the between-group differences.

Difference was observed in the NLRs, MLRs, or the percent of lymphocyte, neutrophil, and monocyte between the multi-mode and RFA groups (Figure 3), suggesting similar baseline preoperative status. To assess whether the therapeutic efficacy of treatment was influenced by the preoperative status, patients were sub-grouped based on preoperative NLR, MLR, or the percentage of lymphocyte, neutrophil, and monocyte (top 50% vs. bottom 50%). As shown in Figure 3, patients with better preoperative status, characterized by lower preoperative neutrophil% (Figure 3C), monocyte% (Figure 3E), or MLRs (Figure 3D), had a longer PFS in patients after from the multi-mode therapy group than those in the RFA group. Furthermore, the PFS of the multi-mode group was not dependent on different preoperative status (Figure 3A–C), while in the RFA group, patients with higher preoperative NLRs (Figure 3A) or neutrophil (Figure 3C), or lower lymphocyte% (Figure 3B) had shorter PFS, indicating disease progression as previously found [13,14]. These results suggested that the multi-mode therapy might alter the patients’ immunosuppressive status to stimulate a more robust systemic antitumor response, thus achieving a therapeutic advantage over the conventional RFA.

### 3.4. Multi-Mode Therapy Promoted Function Maturation of DCs

**Figure 4:**
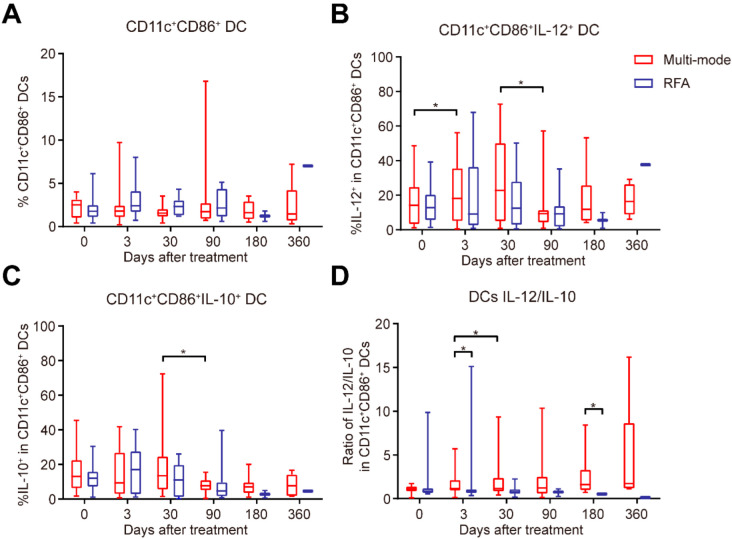
Changes in interleukin (IL)-12 and IL-10 expressions by the CD11c^+^CD86^+^ DCs in patients treated with the multi-mode thermal therapy or conventional radiofrequency ablation (RFA). (**A**–**D**) The proportion of CD11c^+^CD86^+^ DCs (**A**), the expressions of IL-12 (**B**) and IL-10 (**C**) in the CD11c^+^CD86^+^ DCs, and the IL-12/IL-10 ratio (**D**) at each time point were analyzed by flow cytometry. All data are shown as the means ± standard deviations (SDs). * *p* < 0.05, the *p*-value indicating the between-group differences.

Dendritic cells (DCs) are a subset of innate immune cells that serve as key mediators of adaptive anti-tumor immunity. The activation of the adaptive immune system requires the maturation of DCs. DCs are to produce cytokines and to activate cytotoxic T cells by tumor-associated antigen presentation to remodel the immunosuppressive environment, thereby inducing a long-lasting antitumor immune response [15]. To investigate whether the multi-mode therapy could stimulate the DC response, the expressions of IL-12 and IL-10 in DCs were examined to determine their maturation status before and in 3 days, 1, 3, 6, and 12 months after treatment. Although no significant change was observed in percentages of DCs over times (Figure 4A), in the multi-mode group, the expression of IL-12 in DCs was upregulated on day 3, while the expression of IL-12 and IL-10 was decreased in 3 months compared with that in 1 month (Figure 4B,C). The IL-12/IL-10 ratio in DCs was upregulated in 1 month compared with that on day 3 (Figure 4D). Particularly, the IL-12/IL-10 ratios on day 3 and in 6 months in the multi-mode group were much higher than those in the RFA group (Figure 4D). These results indicated that the multi-mode therapy could promote DC maturation at the early stage and maintain their mature function in the long term.

### 3.5. Multi-Mode Therapy Activated the Adaptive Immune Response

**Figure 5:**
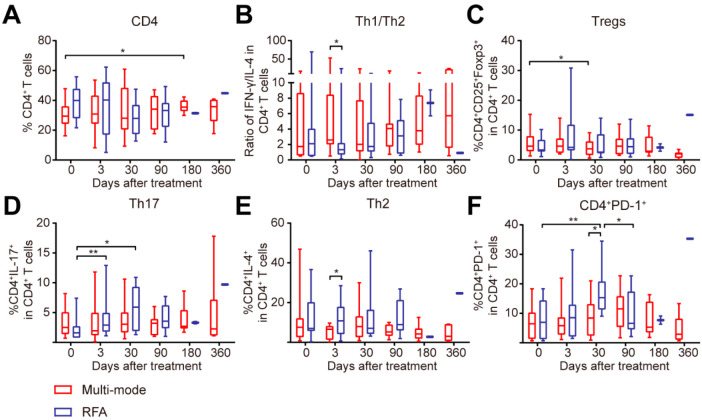
Changes in CD4^+^ T cell subsets in patients treated with multi-mode and radiofrequency ablation (RFA) therapy. (**A**–**F**) The proportion of CD4^+^ T cells (**A**), the ratio of Th1/Th2 subsets (CD3^+^CD4^+^IFN-γ^+^/CD3^+^CD4^+^IL-4^+^) (**B**), the proportion of Tregs (CD3^+^CD4^+^CD25^+^Foxp3^+^) (**C**), the proportion of Th2 subsets (CD3^+^CD4^+^IL-4^+^) (**D**), the proportion of Th17 subsets (CD3^+^CD4^+^IL-17^+^) (**E**), and the expression of PD-1 on CD4^+^ T cells (CD3^+^CD4^+^PD-1^+^) (**F**) were analyzed by flow cytometry. All data are shown as the means ± standard deviations (SDs). * *p* < 0.05, ** *p* < 0.01, the *p*-value indicating the between-group differences.

**Figure 6:**
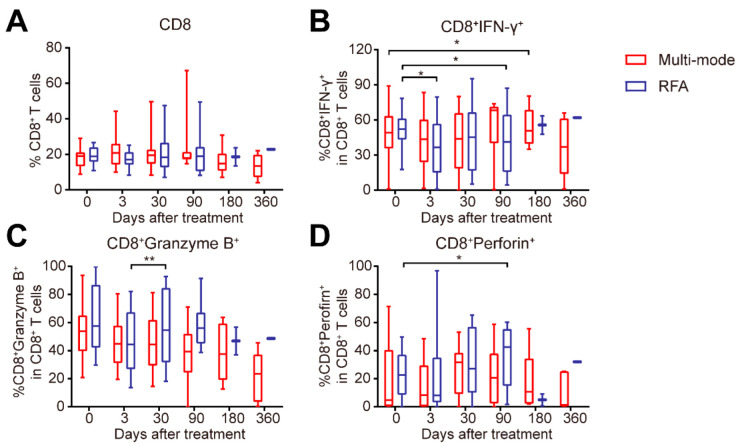
Changes in CD8^+^ T cell subsets in patients treated with multi-mode and radiofrequency ablation (RFA) therapy. (**A**–**D**) The proportion of CD8^+^ T cells (**A**), the expression level of interferon (IFN)-γ (B), Granzyme-B (**C**), and Perforin (**D**) were analyzed by flow cytometry. All data are shown as the means ± standard deviations (SDs). * *p* < 0.05, ** *p* < 0.01, the *p*-value indicating the between-group differences.

Considering the role of adaptive immunity in anti-tumor responses, and T cells playing a crucial role in adaptive immunity against cancer, we further analyzed the proportion of CD4^+^ and CD8^+^ T cells in peripheral blood after the treatments. The multi-mode therapy increased the proportion of CD4^+^ T cells in 6 months (Figure 5A). The Th1/Th2 ratio on day 3 was much higher than that in the RFA group (Figure 5B). The percentage of Tregs was downregulated in 1 month after the multi-mode therapy (Figure 5C). These results suggested that the new therapy could induce CD4^+^ Th1-dominant immune response and decrease the immunosuppressive Tregs. Furthermore, the expression of IFN-γ in CD8^+^ T cells was also increased by the therapy in 6 months (Figure 6B), indicating the activation of CD8^+^ T cells.

CD4^+^ T cells subsets after RFA were also analyzed. As shown in Figure 3A, on day 3, the proportion of Th2 subset in RFA-treated patients was much higher than that in the multi-mode group (Figure 5D). Meanwhile, the proportion of Th17 subset was upregulated on day 3 and in 1 month after RFA (Figure 5E). The expression of programmed cell death-1 (PD-1) in CD4^+^ T cells was increased in 1 month, and was much higher than that in the multi-mode therapy group (Figure 5F). The proportion of CD8^+^ T cells showed no changes in both groups (Figure 6A). The expression of IFN-γ in CD8^+^ T cells was downregulated on day 3 and in 3 months after RFA (Figure 6B), suggesting the suppression of CD8^+^ T cell activation. The expressions of granzyme-B and perforin in CD8^+^ T cells were upregulated in 1 month and 3 months (Figure 6C,D), which might be due to the increased tumor burden in patients as the median follow-up time after RFA was 3.4 months (Figure 2B,C). Overall, these data showed that the multi-mode therapy activated the adaptive immunity, while the conventional RFA might induce CD4^+^ T cell dysfunction in the long term.

## 4. Discussion

The newly proposed multi-mode thermal therapy was well-tolerated and successfully performed in CRCLM patients, with significantly improved PFS regardless of the preoperative status. Further analyses showed that the new therapy promoted the functional maturation of the DCs, increased Th1/Th2 ratio at the early stage, decreased proportion of Tregs, and maintained high level of CD4^+^ T cells at the later stage. In comparison, the conventional RFA increased the immunosuppressive Th2 and Th17 subsets, upregulated PD-1 expression on CD4^+^ T cells, and impaired the activation of CD8^+^ T cells.

Lymphocytes, which are key immune cells in both humoral and cellular antitumor responses, play critical roles in controlling the growth and metastasis of tumor cells [16]. Meanwhile, neutrophils and monocytes can generate an immunosuppressive tumor microenvironment to promote cancer progression through the synthesis of chemokines, pro-tumor growth factors, and proteases [17]. A high NLR or MLR reflects the favor of neutrophils/monocytes over lymphocytes. This indicates the reduction in lymphocyte-mediated immune responses, and impaired antitumor immunity [13,14]. In this study, the therapeutic efficacy of RFA was dependent on better preoperative immune status, characterized as lower neutrophil and monocyte levels and high lymphocyte levels. Conversely, the multi-mode therapy was able to improve the prognosis in CRCLM patients with poor preoperative status, remodeling the tumor microenvironment.

The priming of the adaptive immune response relies on DC activation [18]. In our previous animal model studies, the multi-mode therapy generated significant damage associated molecular patterns (DAMPs) in situ. The release of DAMPs into the circulation promoted the DC maturation and M1 macrophage polarization, triggering the systemic innate and adaptive anti-tumor immunity [19,20]. DCs are the central regulators of adaptive immune response [21]. Mature DCs promote the differentiation of Th1 via IL-12 [22], while IL-10 limits Th1 polarization and results in the imbalance of Th1/Th2 [23]. Moreover, IL-10 secreted by DCs could break the immunological tolerance by Treg induction [24]. In this study, the multi-mode therapy promoted DC maturation and up-regulated the ratio of IL-12/IL-10, which suggested that the therapy could improve the immunological environment in patients.

CD4^+^ Th1 cells have been credited with antitumor activity [25]. IFN-γ secreted by CD4^+^ Th1 cells can upregulate the expression of major histocompatibility complex Ion tumor cells and directly kill tumor cells [25]. Meanwhile, CD4^+^ Th1 can activate antigen-presenting cells (APCs) and maintain the effect of tumor-specific CD8^+^ T cells [26]. Conversely, CD4^+^ Th2 subsets primarily elicit pro-tumorigenic effects [27]. CD4^+^ Th1 cells can inhibit the polarization of Th2 cells [28]. The balance of Th1/Th2 is associated with the outcome of cancer treatment [29]. In this study, the multi-mode thermal therapy increased the ratio of Th1/Th2 on day 3 after the therapy, which could be responsible for the subsequent CD4^+^ T cells differentiation, and the maintenance of IFN-γ expression in CD8^+^ T cells. On the contrary, Tregs are considered to be immunosuppressive and promoted cancer development [30]. A high number of Tregs were associated with poor outcomes in CRCLM, and vice versa [31,32]. In this study, the proportion of Tregs was downregulated in 1 month after the new therapy, which would contribute to the better PFS. However, in colorectal cancer, IL-17 may trigger the release of pro-tumorigenic factors by tumor cells and tumor-associated stroma [33]. The infiltration of Th17 was correlated with poor prognosis [34]. Thus, the upregulation of Th17 at the early stage may be responsible for the shorter PFS after RFA.

## 5. Conclusions

In conclusion, results of this pilot study clearly demonstrated the therapeutic effect of the multi-mode thermal therapy for treating colorectal cancer liver metastasis. The therapy triggered more robust CD4^+^ T cell-mediated antitumor responses and thus improved PFS in CRCLM patients treated. A larger RCT is ongoing to further explore its clinical applications.

## Figures and Tables

**Table 1:** Reagents Used in Flow Cytometry Analysis.

Reagents	Catalog Number
True-Nuclear transcription factor buffer set	424401
Fixation buffer	420801
Intracellular staining permeabilization wash buffer	421002
Cell activation cocktail (with Brefeldin A)	423304
Human TrueStainFcX	422302

All reagents were purchased from Biolegend (San Diego, CA, USA).

**Table 2:** Antibodies Used in the Flow Cytometry Analysis.

Fluorescence Labeling	Antibodies	Clone	Catalog Number
Fluorescein isothiocyanate (FITC)	CD3	OKT3	317306
Alexa Fluor 488	IL-12/IL-23 p40 ^1^	C11.5	501816
PE	Foxp3	206D	320108
	CD11c	3.9	301606
Peridinin-chlorophyll proteins-Cyanine5.5 (Percp-Cy5.5)	CD4	RPA-T4	300530
Phycoerythrin-cyanine7 (PE-Cy7)	CD8a	HIT8a	300914
	IL-4	MP4-25D2	500824
APC	CD25	BC96	302610
Alexa Fluor 647	Granzyme-B	GB11	515406
	IL-10	JES3-9D7	501412
	IL-17A	BL168	512310
Allophycocyanin-cyanine7 (APC-Cy7)	Perforin	dG9	308128
Brilliant Violet 421	IFN-γ ^2^	4S.B3	502532
	CD86	IT2.2	374212
Brilliant Violet 510	PD-1 ^3^	EH12.2H7	329932

All reagents were purchased from Biolegend (San Diego, CA). ^1^ IL, interleukin; ^2^ IFN, interferon; ^3^ PD-1, programmed cell death 1.

**Table 3:** Characteristics of Patients Included.

Characteristic	Multi-Mode Group	RFA Group	*p*
Number of patients	17	14	-
Age	59.88 ± 13.74	64.29 ± 10.22	0.33
Gender (M/F) ^1^	14/3	9/5	0.41
Chemotherapy history (yes/no)	14/3	14/0	0.23
Interval from initial diagnosis to enrollment (days)	679 (72–1675)	819 (250–2738)	0.53
Number of lesions (mean, range)	1.29 ± 0.99	1.64 ± 1.22	0.38
Size of lesions (cm)	2.31 ± 0.97	2.53 ± 0.96	0.66

^1^ M, male; F, female.

## Data Availability

The data that support the findings of this study are available from the corresponding author upon reasonable request.

## Supplementary Materials

The following supporting information can be downloaded at: https://www.mdpi.com/article/10.3390/biomedicines10020280/s1, Figure S1: Gating strategy for immune cell subpopulations. Flow cytometric gating strategy to identify DCs, CD4^+^ T cell subsets and CD8^+^ T cell subsets through surface marker and cytokine expression.
