# Supplementary material for: A Novel Multi-Mode Thermal Therapy for Colorectal Cancer Liver Metastasis: A Pilot Study
Source: Biomedicines. 2022 Jan 26;10(2):280. doi: 10.3390/biomedicines10020280 (PMC8869583; doi:10.3390/biomedicines10020280)
Supplement: Supplementary file 1 [file biomedicines-10-00280-s001.zip › biomedicines-1556204-supplementary.pdf]

Article

Supplementary Materials

A Novel Multi-mode Thermal Therapy for Colorectal Cancer Liver Metastasis: A Pilot Study

Wentao Li[1, 2#], Yue Lou[3#], Guangzhi Wang[3], Kangwei Zhang[3], LiChao Xu [1, 2], Ping Liu [3,*] and Lisa X. Xu [3,*]

1 Department of Interventional Radiology, Fudan University Shanghai Cancer Center, Shanghai, China

2 Department of Oncology, Shanghai Medical College, Fudan University, Shanghai, China

3 School of Biomedical Engineering and Med-X Research Institute, Shanghai Jiao Tong University, Shanghai, China

* Correspondence: Lisa X. Xu, Chair Professor in Biomedical Engineering, AIBME Fellow, ASME Fellow, IAMBE fellow, email: lisaxu@sjtu.edu.cn; Ping Liu, Professor in Biomedical Engineering, email: pingliu@sjtu.edu.cn

# These authors contributed to the work equally and should be regarded as co-first authors

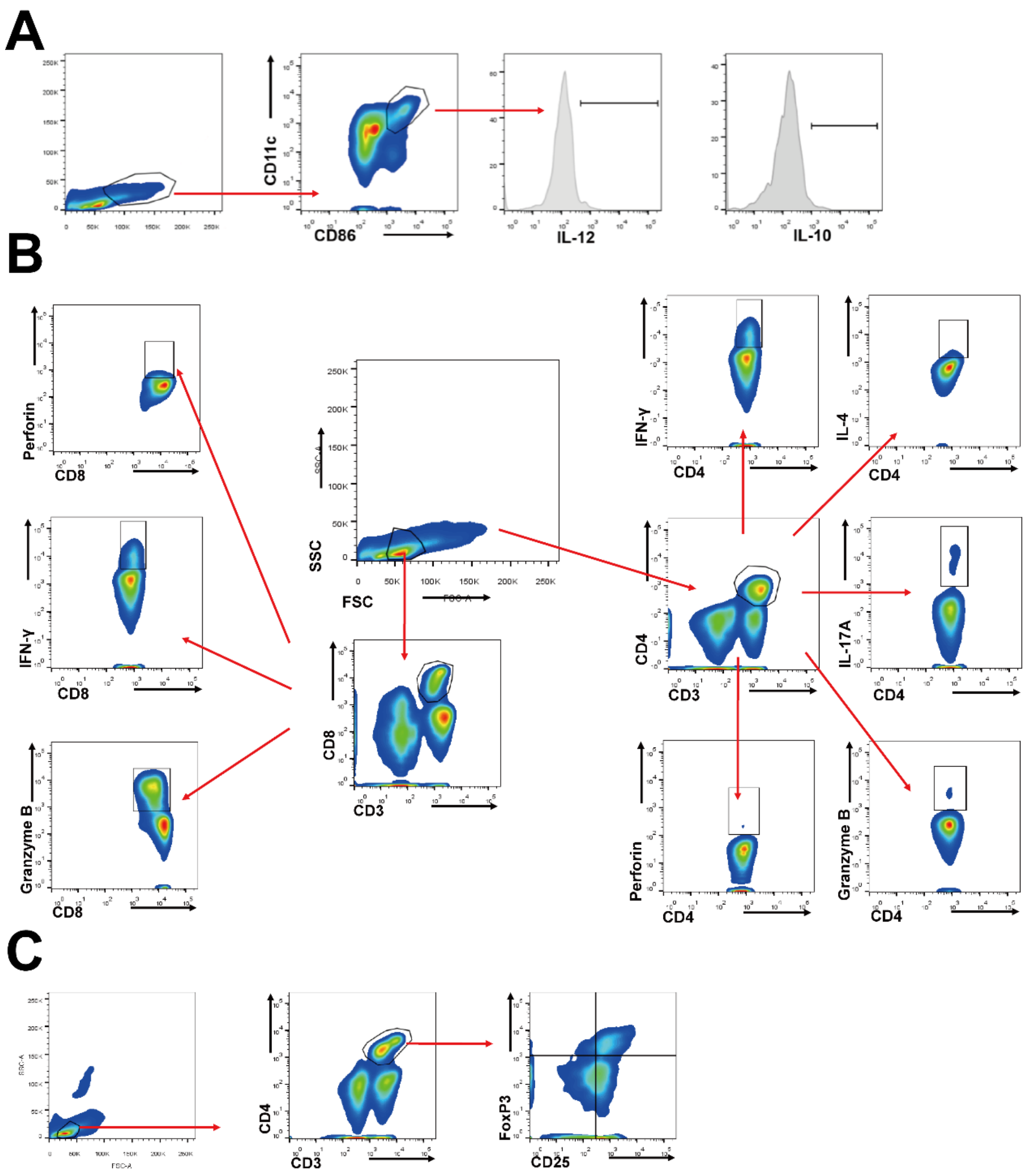

Figure S1. Gating strategy for immune cell subpopulations. Flow cytometric gating strategy to identify DCs, $CD4^{+}$ T cell subsets and $CD8^{+}$ T cell subsets through surface marker and cytokine expression.
